# Design of a Soil Cutting Resistance Sensor for Application in Site-Specific Tillage

**DOI:** 10.3390/s130505945

**Published:** 2013-05-10

**Authors:** Juan Agüera, Jacob Carballido, Jesús Gil, Chris J. Gliever, Manuel Perez-Ruiz

**Affiliations:** 1 Rural Engineering Department, University of Córdoba, Campus de Rabanales, Edif. Leonardo da Vinci, Ctra. Nacional IV- km 396, 14014 Córdoba, Spain; E-Mails: jaguera@uco.es (J.A.); jacob.carballido@gmail.com (J.C.); mc1giroj@uco.es (J.G.); 2 Biological and Agricultural Engineering, University of California, Davis, One Shields Ave, Davis, CA 95616, USA; E-Mail: cjgliever@ucdavis.edu; 3 Aerospace Engineering and Fluids Mechanics Department, University of Seville, Ctra. Sevilla-Utrera km 1, 41013 Seville, Spain

**Keywords:** soil cutting resistance map, site-specific management, soil sensor, GNSS

## Abstract

One objective of precision agriculture is to provide accurate information about soil and crop properties to optimize the management of agricultural inputs to meet site-specific needs. This paper describes the development of a sensor equipped with RTK-GPS technology that continuously and efficiently measures soil cutting resistance at various depths while traversing the field. Laboratory and preliminary field tests verified the accuracy of this prototype soil strength sensor. The data obtained using a hand-operated soil cone penetrometer was used to evaluate this field soil compaction depth profile sensor. To date, this sensor has only been tested in one field under one gravimetric water content condition. This field test revealed that the relationships between the soil strength profile sensor (SSPS) cutting force and soil cone index values are assumed to be quadratic for the various depths considered: 0–10, 10–20 and 20–30 cm (r^2^ = 0.58, 0.45 and 0.54, respectively). Soil resistance contour maps illustrated its practical value. The developed sensor provides accurate, timely and affordable information on soil properties to optimize resources and improve agricultural economy.

## 1. Introduction

Crop yield variability within a field depends on soil properties and environmental conditions. To optimize the management of agricultural inputs according to site-specific needs, geo-referenced information about the site is required [1]. Spatial yield variations provide opportunities for exploring the cause with site-specific technology [2]. An important aspect of precision agriculture has been the use of sensor data to obtain accurate information that help minimize crop yield variation. This georeferenced data is incorporated with more broadly related information such as edaphic, meteorological, biological, anthropogenic and topographic factors. Site-specific management is extremely complicated because all of these factors must be considered. Researchers and farmers must overcome several challenges, such as simplifying the complexities that delineate site-specific management zones based on a single factor (for example, edaphic properties), and determining the yield variation related to this factor [3]. Mouazen and Ramon [4] investigated the use of an on-line measurement system of soil compaction, the bulk density model, in different soil textures (*i.e.*, loamy sand, loam, silt loam and silt).

Some soil parameters vary in space and time. Therefore, logistical and analytical costs are often limiting when addressing spatial soil variability, especially in large-scale applications [5]. The traditional method of exploring field soil variation is through grid sampling, which is time-consuming, labor-intensive and costly. Advanced technologies and developments in precision agriculture applications have allowed researchers to scrutinize an on-the-go soil strength profile sensor [6–8].

To determine the magnitude of the overall compaction or the depth location of the compacted layers, soil strength profile sensors have been developed. Hemmat *et al.* [9] reviewed and analyzed soil profile sensors that should be capable of accurately mapping both spatial and vertical variation in soil mechanical resistance. In this work, two different approaches, a tip-based and a tine-based sensor, were used to classify the soil profile sensors.

With tip-based sensors, soil compaction is traditionally analyzed by measuring soil strength indices such as the cone index (CI). This index is often measured using an American Society of Agricultural and Biological Engineers (ASABE) standard cone penetrometer. The force per unit area required to push the penetrometer through a specified small increment of soil depth is measured. However, a cone index (CI) is a point measurement that exhibits high variability, requires a significant amount of manpower and is time-consuming to measure because large amounts of data are needed to map a field. Current guidelines in the ASAE Standard EP542 [10] recommend that the sample size be based upon visible heterogeneity and that a sample size of at least 20 samples be used to characterize a site. Geo-statistical analysis has proved to be useful for characterizing soil spatial variation properties [6,11,12]. Work at the Department of Biological and Agricultural Engineering at UC Davis has indicated that variation in water infiltration rates caused by soil compaction variability within a processing tomato field was a major factor affecting tomato yield [13].

Over the last few years, interest in applying tine-based sensors for commercial purposes has risen. Their proposed applications can be classified into two types: (1) using an array of strain gauges mounted on a rigid tine [14,15] and (2) multiple active cutting edges [16]. An instrumented implement has also been developed using a load cell to determine soil resistance in real-time to enable field mapping [17]. This system produced satisfactory results but only at one depth, which is a major constraint. Hall and Raper [18] developed equipment consisting of a novel sensor mounted on the leading edge of a tine and a reciprocating drive for oscillating the tine vertically while it moved horizontally through the soil. In this work, 30 sensing tips were used and a wedge index defined as the measured force divided by the area of the tip was used to represent soil cutting strength. By increasing the base area of the tip from 6.25 to 25 cm^2^, the slope of the wedge index and the CI related to the base area increased from 1.52 (*r*^2^ = 0.65) to 2.99 (*r*^2^ = 0.83). These results indicate that a direct equation describing the relationship between the wedge index and the CI might not be possible. This was due to empirical measurement methods that may be affected differently by various soil factors.

Based on previous work [8], Andrade-Sánchez *et al.* [12] developed a soil cutting force profile sensor that consisted of five 5.1-cm long, active cutting elements directly connected to five customized octagonal ring load-sensing units that could measure the cutting resistance of soil directly ahead of the cutting element. This device was capable of measuring soil cutting resistance over the depth profile of 7.5 to 45.7 cm. These load-sensing units were custom-designed based on their relative location along the depth and expected load at that depth to maintain similar sensitivity levels among all five sensing units. A sub-meter accuracy Differential Global Positioning System (DGPS) receiver that used a coastguard beacon differential correction was employed with this system to provide position information. In addition, radar (model Radar II, Dickey-John Corporation, Auburn, IL, USA) was employed to measure ground speed. The effect of travelling speed on the cutting force was not significant between 0.65 and 1.25 m·s^−1^, and the sensor output could be expressed as a function of CI and operating depth with a coefficient of multiple determination of 0.985 [8].

The dynamic effects of an on-the-go sensor moving through the ground can include both inertial forces, due to soil volume acceleration, and changes in ground strength at a high rate of shear. These effects were studied in detail by McKyes [19], who also indicated that the effect of the shear rate was not significant in simply frictional soils but was significant in clay soils and outweighed the inertial forces. The soil force on a tool is known to approximately increase with the square of its speed [20,21].

In the last decade, the integration of Global Navigation Satellite Systems (GNSS) with sensors for off-road vehicle systems and other platforms has provided real-time sub-meter to centimeter-level accuracy and significantly enhanced the spatial accuracy of data needed for precision agriculture [22]. The GNSS receivers are a key part of the precision agriculture technologies, as position information is a prerequisite for site-specific crop management. However, researchers believe that not all of the tasks that are or can be performed in precision agriculture require the same level of GNSS accuracy [22,23]. Some precision agriculture applications, such as yield monitoring, soil samples or variable rate applications, are performed sufficiently accurately with differential GPS (DGPS) devices with submeter accuracy. Currently, Real-time Kinematic-Global Positioning System (RTK-GPS) technology offers the possibility of transitioning site-specific techniques from sub-meter-level precision to centimeter-level precision. Although differential correction signals (DGPS) have been used to successfully geo-position electromagnetic induction (EMI), elevation or compaction soil measurements, accuracies of ±10 cm should be insufficient. Given the topography, the travel direction and terrain irregularities/inclinations, sensor measurements should be corrected. Accurate measurements (±2 cm) of the terrain elevation for DEM construction and geo-referencing geophysical measurements allow for sensor error corrections.

The overall objective of this research was to develop a soil strength profile sensor equipped with RTK-GPS technology that could perform measurements continuously and efficiently at various depths while traversing the field. An articulated parallel linkage system was used to transmit the cutting resistance from the blades to the load cell situated above-ground, which permitted a reduction in the width of the blade and associated energy requirements in soil cutting compared to previously reported research and makes it possible to use this sensor in non-till farms. The specific objectives of this research were:

1. Design and construct a field-ready strength profile sensor.
2. Perform laboratory and preliminary field tests to optimize the sensor for reliable operation.
3. Obtain georeferenced soil mechanical resistance from commercial field and produce soil strength profile variability maps.

## 2. Materials and Methods

We have designed and built a sensor that quantifies the soil cutting resistance free from the influence of the friction force exerted by the ground upon steel blades. The cutting force was simultaneously obtained at different depths. These measurements were performed by making a continuous cut through the soil with four steel blades positioned one behind the other. Each steel blade was at a different depth with narrow cutting widths. In this work, the sensing mechanism, which utilized an RTK-GPS receiver to locate the soil cutting resistance data, was pulled by a conventional tractor and was successfully operated in the laboratory and in a commercial field in Spain. This sensor was specifically designed to be economically feasible for variable-rate management compared to a cone penetrometer grid-sampling sensor [24].

### 2.1. Sensor Description

**Figure 1.**
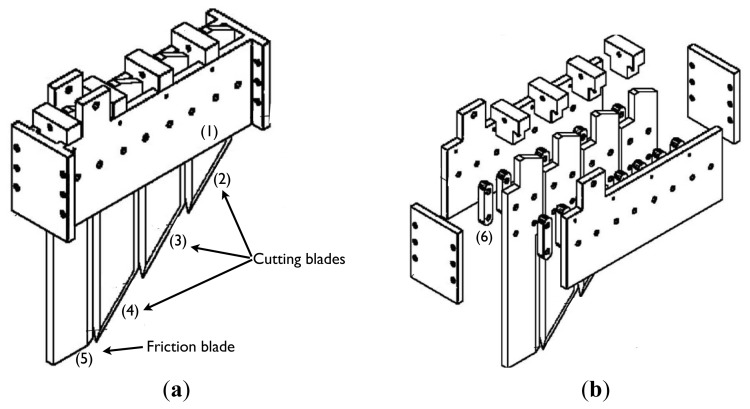
The soil strength profile sensor.

Four blades were each equipped with load sensors (Figure 1(a,b)) similar to the prototype developed by Siefken *et al.* [25], except the load cells that supported the blades were situated above ground. An implement frame was designed, developed and assembled to ensure that the steel blades were orientated vertically during the operation. The cutting blades were located between two horizontal plates (1) of the frame. Vertical support bars (6) were mounted on each side of the cutting blades (2), (3), (4) and the friction blade (5) according to Adamchuk *et al.* [15] and allowed quadrilateral articulation. Each blade module consisted of four vertical bars, two on each side, thus providing mechanical strength. The blades were chamfered around their edges; therefore, the cutting area was oblique (45°) to the soil surface with a blade width of 100 mm. Each blade was 100 mm longer than the preceding blade, except for the last blade. We selected the width of the blade as 10 mm to provide minimum soil disturbance and energy consumption (minimum cutting width). The blades were attached to the implement frame using a shear bolt mechanism, and the implement was attached to the tractor with a three-point hitch. In the working position, the frame is horizontal, and the first blade is at a depth that positions its top most oblique front cutting edge at the soil surface.

### 2.2. Idealized Force and Moment Balance on the Cutting Blade

The portion of the steel blade below the ground surface is subjected to a resultant force (F_R_) that originates from two different mechanisms when the system is moved forward:

1. The cutting resistance is caused by shearing soil aggregates. This force is distributed along the cutting edge. Their resultant (F_C_) is a horizontal vector and is located at a depth that is intermediate between both ends. F_C_ would be located at the midpoint just when the soil exerts a uniform shear throughout the cut edge profile.
2. Frictional force is caused by soil particles pressing on the sides of the blade. The frictional force (F_F_) will be a horizontal vector and is located at a depth that depends on the distribution of these elemental forces. For a uniform distribution, the force vector will be at a depth that divides the exposed surface of the blade into two equal portions.

The F_R_ is in opposition to the advancement of the blade and will be located at a depth that results from the distribution of both components. In addition, this distribution may differ according to the location within the plot of interest.

**Figure 2.**
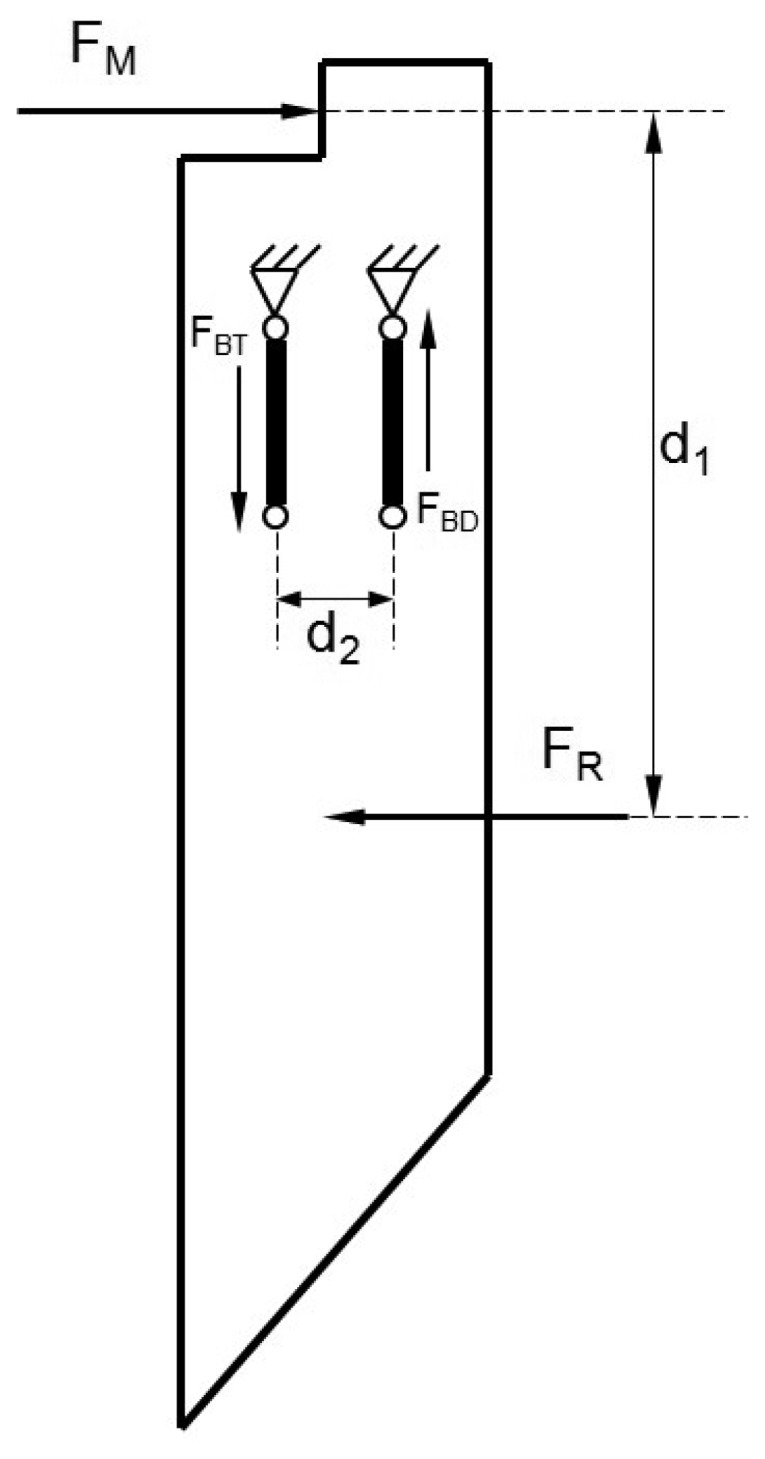
Distribution of F_R_, F_M_ and the distances on the cutting blade.

The reaction force (F_M_), which counteracts the F_R_, was measured by a load cell (model LFH-71/0280 model, Sensotec, Columbus, OH, USA). As shown in Figure 2, the distance between both forces generates a moment that must be counteracted so that the system is in balance. The balance condition causes torque to be exerted on the hinge points of the vertical arms of the blades. The arm located at the front, according to the forward direction, exerts a force on the blade vertically upwards (F_BD_), while the rear arm exerts a downward vertical force (F_BT_). Both must be equal but in opposite directions to cause a moment equal to the product of F_BD_ by the distance that separates them.

Equation (1) illustrates the horizontal forces in balance:

(1) $${\vec{\text{F}}}_{\text{M}}=-{\vec{\text{F}}}_{\text{R}}$$

Furthermore, to maintain the moment balance, the torque generated by the horizontal forces should be equal and opposite to that generated by the vertical forces. This indicates that the force measured by the load cell F_M_ will always equal to the sum of the cutting and friction components, regardless of the depth at which its action is located within the line. The origin is the moment generated by the equal and opposite vertical forces, F_BD_ and F_BT_, the magnitude of which varies according to F_R_ and d_1_:

(2) $$\left|{\text{F}}_{\text{BD}}\right|=\left|{\text{F}}_{\text{R}}\right|\;\cdot \;\frac{{\text{d}}_{1}}{{\text{d}}_{2}}$$

The load cell supporting the blade was situated above ground, thereby reducing the incidence of complications compared to its placement below ground level. The novelty of this sensor is the unique load cell-mediated fastening of the steel blade to its frame support. This allows the load cell to experience horizontal thrust while supporting the blade, thus removing the need to correct the load cell readings to determine the total soil resistance.

To determine both F_C_ and F_F_, a special knife-tool (friction blade) was used. This friction blade does not cut when moving through the soil. It only moves inside the space previously opened by cutting blades and therefore is only subjected to F_F_. The force measured by the load cell of this blade, divided by the ground contact surface, yields the F_F_ per unit area.

The F_F_ value can be applied to the cutting blades, considering its contact surface, revealing what part of the total force, as measured by the load cell, corresponds to the friction and which part corresponds to the soil cutting. Evaluating both forces is an important innovation of this novel strength profile sensor. For site-specific tillage application, only the cutting force is relevant. However, the friction force auxiliary measurement is essential for correcting the total force F_M_ obtained by each load cell attached to the three cutting blades.

### 2.3. Laboratory Tests and Initial Field Tests

Laboratory tests were performed to compare the forces exerted on the blades and the load cell that were installed in the main frame with the force measured by a reference load cell (SM-5000 N model, Interface Inc., Scottsdale, AZ, USA). The reference load cell was connected to the blades through tension locks with hook and eyelet attachments. Both load selections were based on the size and design of the sensing element and on previous experience gained through obtaining the expected maximum soil resistance values with the soil cone penetrometer. Force data for each load cell were conditioned and recorded by a data acquisition system (DEWETRON, Graz-Grambach, Austria). This system is a portable unit compatible with plug-in signal conditioning modules with selectable ranges and an analogic filter that facilitates a suitable signal-to-noise ratio. Load cell and other sensors, such as an accelerometer and thermocouples, can be directly interfaced with the data acquisition system.

The laboratory tests were performed in four replicates. Each blade was tested individually and at different depths (first blade at 10 cm; second blade at 10 and 20 cm; third blade at 10, 20 and 30 cm). Readings for each blade and its depth were collected individually. The reference load cell was linked to the blades through tension locks with a hook and a 50-cm-long eyelet. One end of the tension lock, a steel “S” fastener was used as an adapter for the cutting and frictional blades. At the other end, a 20-cm-long tension lock was attached to a metal pole with sufficient bearing capacity. Increases of 490 N were achieved by manually tightening the tensor lock.

**Figure 3.**
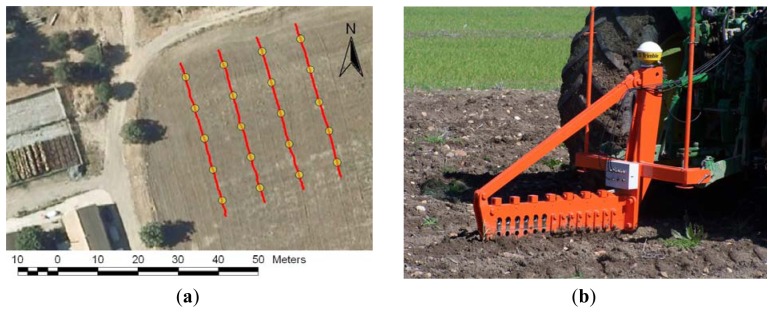
(**a**) The yellow circles represent the geospatial location of each six set of cone penetrometer measurements and the red track the straight strength sensor measurement transect. (**b**) Implement sensor at the working location with a GPS antenna.

Simple field examinations were performed on a commercial field located in the South of Spain to assess adequate sensor performance (Figure 3(a)). Two types of monitors were used: a hand-operated soil cone penetrometer (CI) and the field-ready soil strength profile sensor (SSPS). Soil moisture was measured in field on the test day. SCPS data at three depths (0–10, 10–20 and 20–30 cm) were collected with a 10-m transect spacing at 40 m in length (11 November 2010). The transect spacing was set according to the shape and dimensions of the field. The soil of the field test, a loamy-textured alluvial soil (45% sand, 45% silt, 10% clay), was classified as a Typic Xerofluvent [26].

On a 10-m interval along each transect, six CI profiles were obtained with a hand-operated soil cone penetrometer equipped with a straight circular stainless steel cone at an angle of 30° fixed on a stainless steel bar according to the ASAE S313.3 standard. Operating parameters were set according to the ASAE EP542 standard, and the penetration speed was set to approximately 3 cm/s. The first reading was collected when the cone base was even with the surface of the soil. The reported CI value was the mean value of the pressure (MPa) identified by the cone as it was inserted into the soil. A load cell measured the force with which the soil opposed penetration, and a potentiometer measured the displacement rate to determine the exact depth location of the force data. The hand-operated soil cone penetrometer was retrofitted with an RTK-GPS receiver (model AgGPS 332, Trimble Navigation Ltd., Sunnyvale, CA, USA). This GPS receiver was interfaced to a field computer (model AgGPS 170, Trimble Navigation Ltd.) to record the location of each sampling point. Thirty measurements were obtained with the hand-operated soil cone penetrometer in clusters of five along of the cutting line.

Figure 3(b) illustrates the recording of soil strength measurements with the implement sensor that is being pulled by a tractor. All of the passes were performed at a velocity of 5.7 km/h. To avoid readings influenced by ground breakage effects, measurements were taken approximately 0.3 m from the cutting line of the sensor. To analyze the data, the 15 strength sensor measurements that were obtained close to the measurements collected at the cone penetrometer measurement locations were used to investigate the relationship.

### 2.4. Data Analysis

The performance of the soil strength sensor was evaluated using laboratory and field tests. In laboratory tests, the mean and standard deviation (SD) of simulated soil resistance from the reference load cell for each blade and depth were determined. A regression analysis was performed to investigate the force transmission system (articulated parallel linkage system) when the cutting force changed along the blade. In a commercial field test, a non-parametric one-sided Wilcoxon-Mann-Whitney procedure was used to compare the soil resistance among independent samples. The field trial data was first corrected in order to obtain the cutting forces, free from the influence of the friction forces, using for that the data from the frictional blade. The relationship between the cone penetrometer measurements and cutting forces was determined using the least trimmed squares regression [27]. Analysis of the dataset was performed with R software [28]. A robust Elliptic Plot using the Replot function was used to detect and study outliers [29]. A geostatistical method of interpolating sparse data for random spatial processes was used to achieve the cutting resistance maps (ordinary kriging). The original formulation of kriging is the most robust method and is often used in precision agriculture [30].

## 3. Results and Discussion

### 3.1. Laboratory Test

Eighty separate static force measurements were collected for each blade and for the reference load. The means and SDs of the static force exerted on the blades by the load cell were calculated in increments of 490 N from 0 to 4413 N. The average SDs for static force for the first, second, third and fourth blades were 4%, 5.3%, 3.4% and 5.2%, respectively. These SDs displayed acceptable performance indices for this method. An SD of 5% or less indicates adequate method performance, whereas an SD of 10% or higher indicates problematic performance.

**Figure 4.**
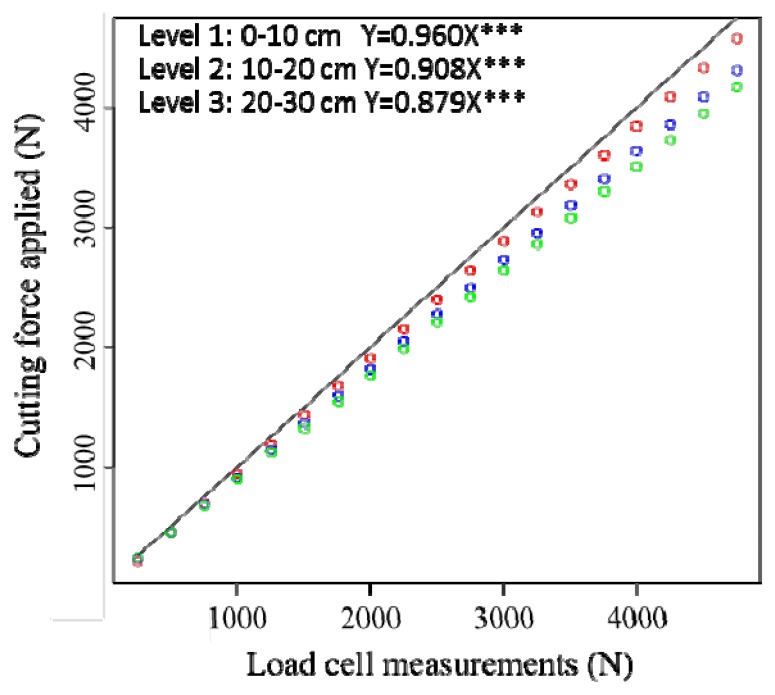
Scatter plot and fitted regression through the origin for the third steel blade. Level 1 (0–10 cm) in red circles, Level 2 (10–20 cm) in blue circles, Level 3 (20–30 cm) in green circles.

The laboratory tests demonstrated that the strength sensor design performed successfully based on an articulated parallel linkage system and separated steel blades. This indicated that the application point of the cutting force is independent of the cell load measurements. Figure 4 displays the regression through the origin that was employed, where *Y* values are the cutting force applied and *X* values are the load cell measurements on the third steel blade. The estimated regression functions are:

The equations and regression plots for the steel blade (1), (2) and (4) were similar. However, we observed minimal slope differences in all of the blades. The trend indicates that when the support is farther from the resultant force (*i.e.*, at a greater depth), the response is smaller. Stated another way, the same F_R_ applied to the blade generates less force on an upper support that is more distant from the support. This is caused by friction at quadrilateral articulation. This friction is proportional to the axial stress that supports the quadrilateral arms, which is proportional to the torque moment that must be balanced. Although the force applied on the blade is the same, the greater the distance from the reaction (upper support), the greater the friction force absorbing articulations, which withstands less force to maintain balance. Therefore, this test is not just a simple calibration but also a way to quantify the influence of friction phenomena in the initial theoretical model that were not taken into account.

### 3.2. Simple Field Examination

A simple field test was conducted to analyze the performance of the sensor as it traversed the ground. The main objective of this brief field test was to demonstrate an adequate performance, with particular attention to problems with operating the equipment (mechanics and electronic components), integrating technology systems and collecting and managing strength force data.

The sensor measurements were compared with the hand-operated soil cone penetrometer measurements. Equation (3) provides insight into the soil cutting force requirement of this sensor. If the SCPS cutting force requirements are linearly related to the soil CI values, then this sensor can be used to measure soil strength. At present, this sensor was only tested in one field under one gravimetric water content condition (θg^0–10^=0.204 g·g^−1^; θg^10–20^=0.184 g·g^−1^; θg^20–30^=0.170 g·g^−1^). In this field examination, the relationships between the SCPS cutting force and soil CI values were assumed to be quadratic for the various depths considered. However, it poorly correlated at each of the depths: 0–10, 10–20 and 20–30 cm (r^2^ = 0.58, 0.45 and 0.54, respectively). Data collected with large handles and a slightly stony ground could produce this type of data interference. This relationship was similar in magnitude to that observed by Chung *et al.* [31], although this study introduced two research fields with variations in bulk density, soil water content and soil texture.

**Figure 5.**
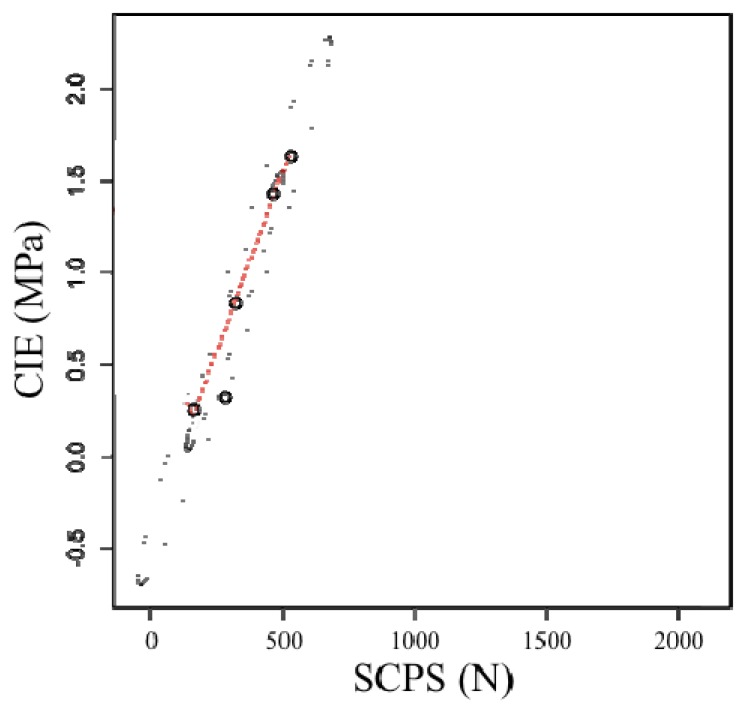
One-pass relationship between the profile-average cone index and the soil cutting resistance.

Profile-average measurements of the cutting force are plotted *versus* measured profile-average soil cone index values in Figure 5. Five data points were compared by each strength sensor pass. As an example, each point in Figure 5 represents the profile-average of six measurements that were collected in clusters of five CI values along 10 m of cutting line. In this study, the average data profiles were analyzed independent of depth. These initial test results were satisfactory but revealed a more general trend, such as the mapping of spatial variability with as many data as possible. In the future, large commercial field tests are needed to verify the potential of this developed soil strength profile sensor.

The relationship between the CI equipment (MPa) and the soil cutting resistance sensor (N) reveal a clear linearity in the measurements taken (*r_xy_* = 0.99, p < 10^−4^). There were no outliers (Figure 5). The linear regression was:

(3) y=−0.4+0.04x

where:

y = CI measurements (MPa)
x = strength profile sensor measurements (N)

**Figure 6.**
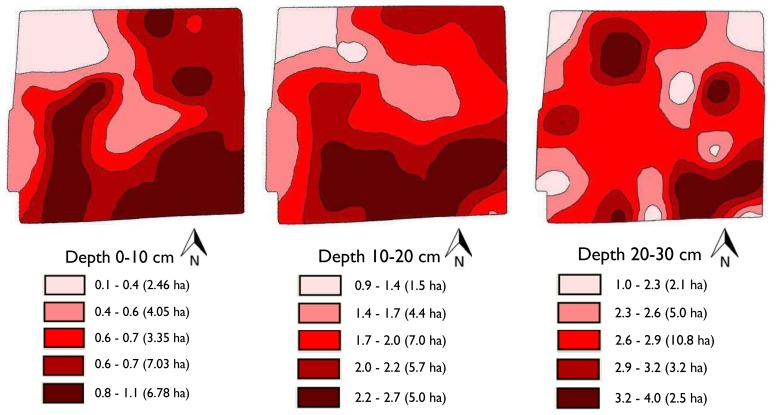
Contour maps of soil resistance (MPa) at different soil depths using SSPS data.

A potential application of this strength profile sensor is site-specific tillage as shown in Figure 6. The performance of this sensor resulted in soil resistance that ranged between 0.1 and 4 MPa. Contour maps were generated using a kriging. We assumed that 2.5 MPa was the agronomically limiting CI value based on prior studies [32]. This indicates that for all measured values of SSPS that exceed this value at a certain depth, the soil should be tilled to that depth. This work demonstrates the possibility of identifying localized areas for sub soiling and the capacity to subsequently till to variable depths to obtain desired soil strength conditions. This study suggests that significant amounts of energy could be saved if site-specific tillage is implemented.

## 4. Conclusions

We developed a prototype SSPS sensor continuous soil cutting resistance mapping at multiple depths while traversing a field and tested it under laboratory and field conditions. Our major contributions are as follows:

– Design of a sensor implement based on an articulated parallel linkage system and separated steel blades, which successfully records the F_R_ independent of the cutting force application point. However, a redesign and redevelopment of several aspects, such as bearing capacity, are needed to avoid variations at different depths.
– Continuous and efficient data collection by a soil strength profile sensor, equipped with RTK-GPS technology, at various depths while traversing a field.
– Assessment of the relationship between the SSPS and CI measurements (r^2^ = 0.58, 0.45 and 0.54) when the data were segmented by different depths (0–10, 10–20 and 20–30 cm)
– Assessment of the relationship between the profile-average measurements of the cutting force and measured profile-average soil cone index values revealed coefficients of determination greater than 0.9 when measured with the soil strength profile sensor.

Use of this innovative sensor for soil cutting resistance mapping may result in a new era of site-specific tillage, which we plan to pursue through future research. Further work is also needed to provide additional insight into the SSPS and CI relationship in large commercial fields so that data obtained with the strength sensor can be related to the plethora of published research that used the CI to quantify soil strength.
